# Sense of place and experimentation in urban sustainability transitions: the Resilience Lab in Carnisse, Rotterdam, The Netherlands

**DOI:** 10.1007/s11625-018-0562-5

**Published:** 2018-04-27

**Authors:** Niki Frantzeskaki, Frank van Steenbergen, Richard C. Stedman

**Affiliations:** 1 Dutch Research Institute For Transitions, Faculty of Social Sciences, Erasmus University Rotterdam, Burgemeester Oudlaan 40, 3000DR Rotterdam, The Netherlands; 2 Human Dimensions Research Unit, Department of Natural Resources, Cornell University, Ithaca, USA

**Keywords:** Sense of place, Experimentation, Cities, Urban transitions, Vision, Sustainability

## Abstract

Experimentation as a means of governance for sustainability transitions has been advocated for years by transition scholars and geography scholars. We propose that examining the impact of experimentation requires an understanding of its embeddedness in place as a socio-spatial context. This notion of embeddedness, which conceptually aligns well with the understanding of sense of place, is under-examined in sustainability transitions literature. By conjoining the sense of place and sustainability transition literatures, we conceptualize that sense of place can be one outcome of experimentation fostering sustainability transitions. We examine urban living labs as an open format of urban experimentation, where multiple actors interact with the aim to co-design, test, and implement governance innovations. From the literature, we have distilled three phenomena that relate to a sense of place as mechanisms for transformation: a symbolic understanding or meaning of place; a narrative of place that connects to a transformative vision; and new types of relations between people and place. With this conceptual lens, we analyze our case study, an urban living lab called The Resilience Lab in a neighborhood of the city of Rotterdam, The Netherlands. Drawing from a longitudinal case study research, we contend that urban living labs can connect a sense of change (transformation) with a sense of place by co-creating new narratives of place, by co-producing knowledge on new practices and new relations between people and place, and by allowing the co-design or (re)establishment of places with symbolic meaning. As such, urban living labs facilitate urban sustainability transitions.

**Electronic supplementary material:**

The online version of this article (10.1007/s11625-018-0562-5) contains supplementary material, which is available to authorized users.

## Introduction

Urban planning and policy experimentation has been showcased over the past years as a means for addressing sustainability challenges. Experimentation as a means of governance for sustainability transitions has been advocated by transition scholars (Smith and Raven 2012; Frantzeskaki et al. 2012, 2014; Wolfram and Frantzeskaki 2016; Pereira et al. 2015; Liedtke et al 2012; Sack 2011 and Sengers and Raven 2015) and geography scholars (Castán Broto and Bulkeley 2014), exploring how incorporating new forms of urban governance may increase the sustainability of urban development programs.

Experimentation can mean many different things. Experimentation is a governance approach of sustainability transitions that entails a multi-actor collaboratively and creatively trialing of new ways of organizing, doing, relating and in this way, generating alternative (forms of) innovative solutions with the potential to address contemporary urban challenges. Our conceptualization aligns with the generative experimentation idea from Ansell and Bartenberger (2016). Experimentation as thus is a solution-oriented approach that allows learning and knowledge sharing and co-production. Our focus here is on “urban living labs” as open format experiments, where multiple actors interact with the aim to co-design, test, and implement governance innovations (Lehmann et al. 2015; Voytenko et al. 2016). Urban living labs are purposefully designed to bring together multiple actors that seek to address contemporary sustainability challenges, foster learning (Bulkeley et al. 2016) and to “encourage sustainable innovation” (Ansell and Bartenberger 2016, p. 68).

Although early research examined urban living labs as test beds for technological innovation, recent work has focused on other forms of innovation including social innovation (Franz 2015; Edwards-Schachter et al. 2012).

By definition, urban living lab experiments take place in a geographical and social setting and are thus, anchored in a particular local context which surrounds the ‘lab’ in question, contributed resources to it, influences its meaning, and in turn is affected by it. A socially centered approach to researching urban living labs thus requires understanding translation and contextualization and increasing inclusiveness and function as spaces of encounter (Franz 2015, p. 58). Emphasizing place can deepen the conceptualization and design of urban living labs, as governance innovation instruments. Accordingly, we propose in this paper that examining the impact of urban living labs in urban sustainability transitions requires an understanding of its embeddedness in place as a socio-spatial context.

The notion of embeddedness can be conceptualized in terms of territory, networks, and societal structures (see Hess 2004); this multi-faceted understanding aligns with sense of place. Sense of place as embodying meanings and attachment (more on this in “Enter sense of place and its transformative potential”) as an outcome of experimentation in an urban living lab is under-examined in sustainability transitions literature. Although there has been an increasing attention to geographic and spatial dimensions—especially cities and nations—within transition literature in recent years (Binz et al. 2014; Coenen et al. 2012; Senger and; Raven 2015), it remains unclear how and to what extent place-embeddedness influences and is influenced by the process and impact of urban experimentation. It is exactly this gap in the literature that we aim to address in this paper. By drawing on the literature on insights from different disciplines (sociology, geography and anthropology) in regard to a ‘sense of place’ (Gieryn 2000; Gieseking et al. 2014; Tuan 1974), we conceptualize that sense of place can be one particular outcome of experimentation that influences urban sustainability transitions by fostering meanings and attachment. Against this backdrop, we examine the following research questions: how does experimentation contribute to creating new sense of place (meanings and attachment) and/or interact with previously held meanings in urban settings, e.g., neighborhoods? If so, how does creating new place meaning affect urban sustainability transitions? What are the implications and design characteristics for experimentation to contribute to urban sustainability transitions that are place-embedded?

## Sustainability transitions and sense of place

### Sustainability transitions and experimentation

Experimentation is central for instigating sustainability transitions (Frantzeskaki et al. 2014; Wolfram and Frantzeskaki 2016; Bulkeley et al. 2016). Previous attention on transition experiments and the settings in which they occur has focused on their effect on sustainability innovations (Sengers et al. 2016), how innovation can be embedded in governance processes (Bettini et al. 2015), and the role partnerships in fostering outcomes (Frantzeskaki et al. 2014). Experimentation has been argued to be a way to navigate urban politics and urban transformation dynamics (Broto and Bulkeley 2013; Nevens et al. 2013), but doing so requires that we pay attention to the embedded geography and place characteristics of experimentation (Coenen and Truffer 2012).

Transition experiments have a “place character” that transcends the technological application, and can create place-specific meanings while building from existing meanings. Sometimes, this is even an objective—i.e., to intervene in place as urban revitalization or regeneration interventions—even if not explicitly stated in place terminology. Experimentation is invoked as a preferred way to innovate urban governance (Burch et al. 2016; Frantzeskaki et al. 2017) and to facilitate shifts of urban programs towards more sustainable outcomes. It has become so prominent in cities that inspired new concepts like the experimental city (Evans et al. 2016).

Bringing in a place perspective helps to move transition experiments beyond systemic-level interventions neglecting the view of the agency outcomes they may bring. Even in conceptual impact frameworks (Luederitz et al. 2016), the creation of agency and/or empowerment of agency in place as an outcome of experimentation are often overlooked. In such instances, place elements are implicit. In our paper, we propose that for urban experimentation, an understanding of the role of place, or, the ways place plays out in mediating between experimentation and sustainability transitions is essential.

### Enter sense of place and its transformative potential

Place, as a core construct in geography (Sack 1980, Relph 1977; Tuan 1977), embeds social meanings and social relations in particular location-based contexts. This contrasts with social theoretic approaches, which sometimes consider social relations, meanings, and the like, as completely de-contextualized, or acting as though they happened “on the head of a pin” (Gieryn 2000). Sense of place, in particular, is “the collection of meanings, beliefs, symbols, values, and feelings that individuals and groups associate with a particular locality” (Williams and Stewart 1998). As stated by Stedman and Ingalls (2014, p. 129), “any place embodies a multiplicity of meanings, some nature-based and some not, although some places exhibit a wider range than others”. Our conception of place (see also Masterson et al. 2017; Stedman 2016) emphasizes the distinction, but interrelation between cognitive meanings (Stedman 2008) and attachment, or an emotional bond, usually positive, between individuals or groups and their environment (Altman and Low 1992; Williams et al. 1992; Jorgensen and Stedman 2001; 2006). Simply put, preferred meanings of place form the basis for attachment: we become attached not simply to the place itself, but to the meanings that we hold for it (Stedman 2002; 2004, Stedman and Ingalls 2014, Steward et al. 2004).

Sense of place can enhance urban resilience via strengthening the connections between people and their environment (Kudyrastev al. 2012). Such interventions have been termed restorative topophilia, which “represents an opportunity for positive dependence that underpins the emergence of virtuous cycles” in urban social–ecological systems, relating to a strong sense of place (Tidball and Stedman 2012, p. 297). It relies on creating or strengthening new community relations with place: because of these relations, new place meanings, characteristics, and capacities, and attachment are regenerated. As thus, topophilia is “constructed” and essentially is important to be restored in ‘red zones’ (Tidball and Krasny 2014), such as urban areas that “have suffered long-term erosion and decline through economic stagnation and the disintegration of meaningful social networks” (Stedman and Ingalls 2014, p. 131). New or changed meanings, despite their power, can also create polarization and thus can facilitate or impede transitions to sustainability (Chapin and Knapp 2015; Masterson et al. 2017; Stedman 2016).

Despite the potential utility of the above, such efforts have not been anchored in the language or methods of experimentation. From transition studies, we contend that transition experiments entail spaces of dialogue and intervention that can help shock the system outside a trap by empowering communities, facilitating dialogues, and actions for moving forward and fostering innovations for sustainability. This corresponds with the understanding of Stedman and Ingalls (2014) who position topophilia “as a powerful base for individual and collective action that repair and/or enhance valued attributes of place” (Tidball and Stedman 2012, p. 297). This way, (re)creating attachment and meanings, can result from a learning process where socially constructed meanings can be solicited and negotiated in urban experimentation settings like urban living labs. Hence, this type of experimentation can ultimately, lead to place transformations.

### Bridging sustainability transitions and sense of place: meanings, narratives, and relations

Bringing the sense of place and sustainability transitions literatures together, in a parallel way to Masterson et al. (2017) integration of sense of place and social–ecological systems, we conceptualize that sense of place can be an outcome of experimentation that may contribute to urban sustainability transitions. With this, we propose a conceptual lens to address the first research question ‘How does experimentation contribute to creating new sense of place in urban settings, e.g., neighborhoods?’ and to use as a heuristic for analyzing our case study.

From the writings on sense of place and sustainability transitions engaged above, we distill that there are three key phenomena that relate to sense of place-based transformations facilitated through experimentation:

#### New relations between people and place and between people in the place

Sense of place is embedded in the socio-physical context. This means that relations between people and places emerge from the materiality of place: what resources are in a specific place (Stedman 2002) are how they are used (Williams 2014). Relations between people and place can thus provide clues to sustainability and capacity for transition/transformation (Stedman 1999). Power relationships that shape historical and current interactions with places are also crucial (Chappin and Knapp 2015; Ingalls and Stedman 2016; Cresswell 1996): some meanings, by virtue of their taken-for-grantedness, become seen as “normal” (Cresswell, Stedman 2016).

Sense of place is also created and recreated through social relations and networks. Urban experiments contribute to new relations of people and place in the sense that “made, unmade and remade in relation with human projects” these relations (Entrikin and Tepple 2006) and further fostering collective relations to place. Experimental settings enable shifting social relations and establishing new ones. Our work, following this argument, will explore whether new relations and diverse networks or coalitions emerge in our empirical case.

#### A narrative of place that connects to a transformative vision

Multiple narratives of place can co-exist (Stedman 2016) showing the potentially contested understandings of place among different members of a community. A narrative of place is the stringing together and communication of these symbolic understandings of place and the related experiences. As Chapin and Knapp (2015, p. 39) argue, “sense of place is often contested and not a simple panacea for stewardship, as sometimes assumed by environmental advocates”. Narratives of place (Russ et al. 2015) can illustrate the multiple understandings and elucidate “the complexity of sense of place”, meaning that it can be both instrumental and detrimental for change (see also Marshall et al. 2012). Individuals and/or communities inspired from the transformative place vision may mobilize and select actions to realize these visions that in turn will transform current place elements to desirable forms.

From the transitions perspective, visions are pivotal for mobilizing, inspiring and attracting action for transformative change (Nevens et al. 2013; McPhearson et al. 2016). What work on experiments from the sustainability transitions studies has been lacking is the connection of a vision narrative to place, albeit a rather a strong focus on values and aspirations of the future. As thus, in our conceptualization of sense of place as catalytic for urban transformations, we seek to discover narratives of place that encapsulate transformative visions or elements of (theories of) change in them (Williams 2014; Russ et al. 2015).

#### A symbolic understanding of place

Symbolic understanding includes elements that capture the sentiment of the community about the place of interest often illustrating the “sense of belonging” and encapsulates multiple local experiences (Wilbanks 2015, p. 76; Tomaney 2014). The meaning of the human experience, the emotions and thoughts accompanying it provide the foundation for place attachment (Stedman 2008) and the narratives of place described above. A plurality of symbolic meanings can co-exist “leading to different attitudes, intentions and actions (…) despite shared appreciation for the same biophysical features” (Chapin and Knapp 2015, p. 41; Stedman 2016).

From the transitions perspective, we align with the sociocultural understanding of place meaning described above. Urban experimentation processes contribute to learning and shifting meanings by socially constructing them through purposeful change, and bringing these meanings to the fore as topics of discussion. Interventions in the place can reinforce or challenge symbols of place identity. Such iconic projects in places can function as place settings that represent the embodied symbolic understanding. Such iconic projects may be implemented with an explicit objective in eliciting to new place meanings (e.g., neighborhood community centers, Medved 2017) or to illustrate as outcomes of experimentation possible trajectories for sustainability or resilience producing new place meanings as ‘positive side effects’.

In a nutshell, place-explicit transition experiments can connect a sense of change (transformation) with a sense of place by co-creating new narratives of place, co-producing knowledge on new practices and new relations between people and place and by allowing the co-design or (re)establishment of places with symbolic meaning.

## Methods

### The case study context: Rotterdam and its urban regeneration agenda

The city of Rotterdam has always been a port city. Traditionally, the port workers from the late 1800s and 1900s settled on the southern banks of the Maas River and closest to the city harbour. With the gradual automation of shipping activity and the move of the port out of the city center, the neighborhoods south of the river began to face increasing hardship. These kind of urban neighborhoods in the Netherlands have been increasingly targeted for ‘revitalisation’. This is especially true in Rotterdam, where the ‘deprived neighborhood’ discourse has been increasingly popular the last two decades. Next to this, strong focus on neighborhoods as sites for a wide range of (economic, political, cultural, etc.) interventions, the municipality of Rotterdam is well known for targeting crime, and its high ambitions regarding livability (Schinkel and Van den Berg 2011, p. 1917).

The southern neighborhood Carnisse, with close to 11,000 inhabitants, is one of the forty most ‘disadvantaged neighborhoods’ in the Netherlands (Ministry of Housing 2007). Carnisse has the lowest average income per year in Rotterdam (€ 23,300 in 2014) and has a relatively old and neglected housing stock. Migration streams are quite high: approximately 55% of the people live less than 5 years in the neighborhood and thus perceived social cohesion is relatively low; Carnisse thus scores poorly on different municipal indexes regarding safety, social cohesion, and housing.

Confronted with these problems, Carnisse has been the target of numerous programs by national and local governments for improving housing, security, schooling and working. These efforts are interwoven with broader developments, such as the economic crisis, a reforming welfare state, and a reinstated neoliberal agenda that calls for a ‘participation society’. Rather than isolated issues, the problems in Carnisse are interlinked and argued to be of a persistent nature.

In recent years, the national neighborhood approach changed and the role of citizens was emphasized, in the direction that citizens should become more active in addressing and solving problems in their living environment (Visitatiecommissie Wijkenaanpak 2011). This shifting understanding is part of a broader discourse on the changing roles of citizens and governments in what came to be known as ‘Big Society’ in the UK (Ransome 2011) and ‘participation society’ in the Netherlands (Putters 2014; Tonkens 2014).

This discursive shift to a participatory society results in a challenging deadlock: in areas where the level of self-organization among local communities is perceived as the lowest, the demand for self-organization to tackle multiple systemic problems becomes the highest (i.e., the more extensive and complex the challenges, the higher the demanded for self-organization and self-resolving capacities). In addressing this challenge, the public sector is increasingly looking for innovative modes of governance.

### Case study: the Resilience Lab in Carnisse, Rotterdam

**Fig. 1:**
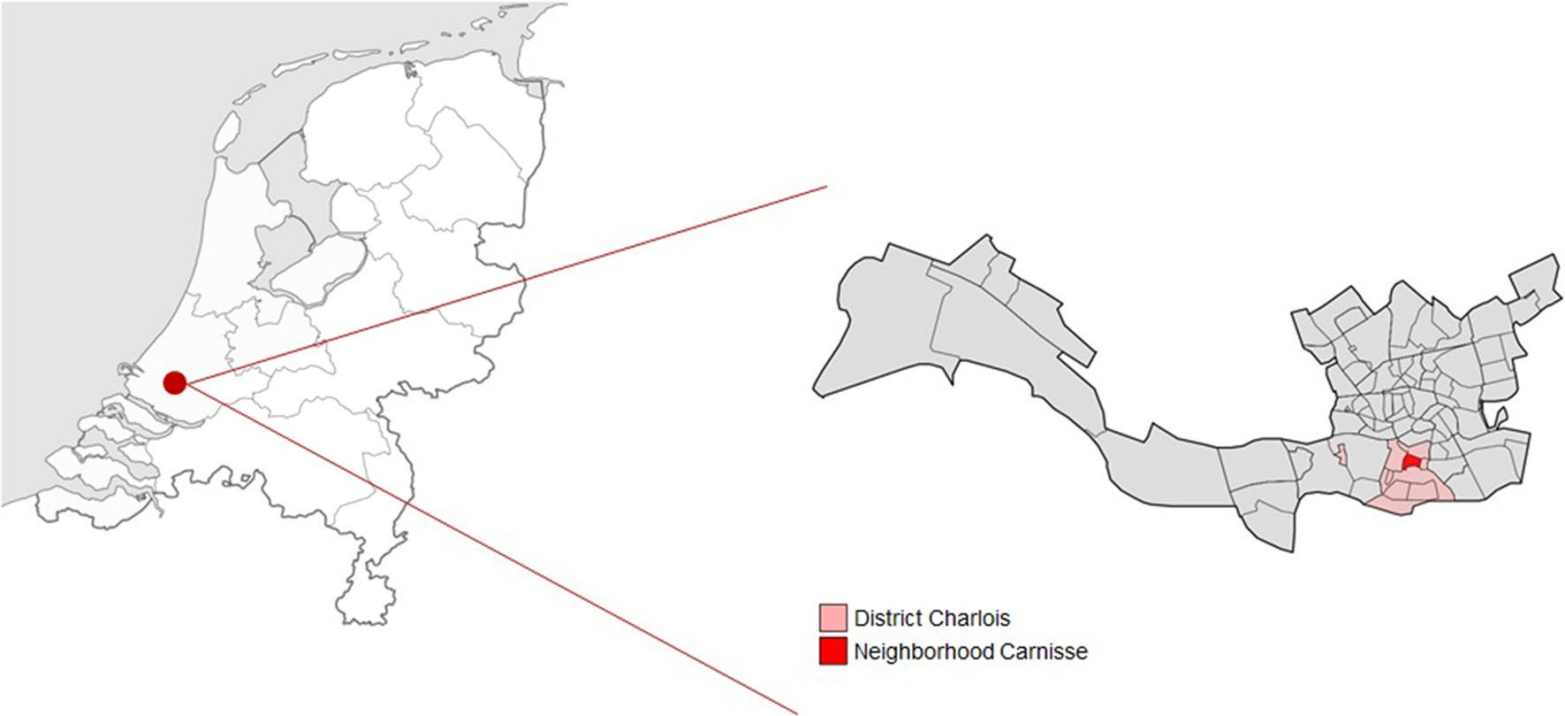
Map of Carnisse. This map zooms in on The Netherlands and shows were the city of Rotterdam is located (left) and on the right a map of Rotterdam is shown were both the neighborhood of Carnisse is located and the greater district of Charlois

We examine the case of a project called Veerkracht Carnisse which is an urban regeneration experiment that focused on empowering local communities and fostering urban sustainability and resilience with a place-making orientation in mind. The case of Veerkracht Carnisse is an urban living lab—i.e., the Resilience Lab in English—and in geographical terms, the primary focus was the neighborhood of Carnisse with secondary focus, the larger district of Charlois (Fig. 1). The Resilience Lab had an official running time of 4 years, starting with a period of concept development and scoping in 2009, and officially initiated in September 2011 and concluded in September 2015. The Resilience Lab was a consortium of four partners: Rotterdam Vakmanstad, Creatief Beheer, Bureau Frontlijn and the Dutch Research Institute for Transitions (DRIFT). The last partner is a research institute where several action researchers were active in Carnisse. What tied these four different partners together was the need to address global transition challenges locally through action, research-community interfaces and collaborative governance. Having been active in different neighborhoods and districts in Rotterdam, they all identified and encountered persistent problems in different societal systems (e.g., education, welfare, health care, and food), which led to occasional projects and sporadic short-term collaborations. From these, the idea of a more extensive and longer term collaboration grew, since the organizations recognized themselves in each other’s societal critique, vision and practices.

In 2009, the concept of experimenting as a means to create and/or shift neighborhood place meanings as part of a large regeneration program was initiated, and discussions with the municipality of Rotterdam started. This proved to be a long process due to contestation from different municipal department and policy makers. In 2011, the program was approved and it eventually started in September 2011, with a running time of 4 years. None of the partners had been active in Carnisse before 2011, although they were active in the larger district of Charlois. Due to this lack of local knowledge, the practitioners of the Resilience Lab engaged local networks through interviews, meetings and collaborations with volunteer organizations, local businesses, and welfare organizations.

During these years, there was a continuous collaboration of the partners in searching for new ways to foster neighborhood resilience. The Resilience Lab activities focused on urban regeneration and specifically on poverty reduction, the upbringing of children, and democratic reform for local development programs. Primary activities were greening the public space by gardening, family educational coaching and assistance with child rearing, introducing philosophy, cooking and judo lessons in schools, and mobilize local communities by envisioning a future Carnisse. The target groups of the Resilience Lab included primarily children (aged 4–12 years), their families, schools (board, teachers and parents) and residents or volunteers actively involved in community life. Also included were the networks in Carnisse and Charlois consisting of professionals, civil servants, social workers, and entrepreneurs.

The main assumption behind the Resilience Lab was that resilient and vibrant communities are needed to better address transition challenges at a local scale. Hence, the Resilience Lab did not seek to shift a certain regime on the neighborhood level, but tried to stimulate certain boundary conditions (awareness, skills, capabilities, social cohesion, etc.) as to address several regime shifts in practice within systems like health care, welfare, food and energy. By experimenting, it wanted to showcase alternative ways of doing, knowing and organizing in practice and show how different transitions become tangible on the level of neighborhoods and communities.

Carnisse has a relatively large private housing sector (about 85%) compared to other neighborhoods: therefore, one of the typical strategies like demolishing aged public housing and engaging with investors like housing associations were not applicable. Consequently, the municipality decided that Carnisse was a suitable place for experimenting with a new form of urban regeneration due to inactivity of local inhabitants and low interest from housing associations in the area. This opened the institutional space for the Resilience Lab that was a test bed for new methodologies and innovative practices.

A long history of policy efforts and participatory processes from neighborhood programs in Carnisse left inhabitants weary of ‘outside’ involvement. Distrust was linked to these projects’ tendency to portray the neighborhood as disadvantaged, an image with which frustrated many locals and in which they did not recognize themselves. Along with the budget cuts, changing responsibilities of government and citizens as well as the erosion of old welfare structures, made Carnisse a challenging context to frame and start an urban living lab. Another challenging feature that participants raised was the relative openness of both the process and outcome, leading to scepticism by local policy makers on the outcomes of the Resilience Lab. To address this, the Resilience Lab had to prove to the residents and other stakeholders in the neighborhood the benefits from being involved in it. This required a deep study of the dynamics of the neighborhood, building networks based on reciprocity and gaining trust over time by showing results that benefitted the local communities. These proved to be conditions for the place-based experimentation of the Resilience Lab. Fast-forwarding to 2015, it becomes apparent that the Resilience Lab has become embedded in the dynamics and characteristics of Carnisse. It is active on several primary schools, community gardens, neighborhood center and families that are routed in the social fabric of the neighborhood and its local communities.

### Longitudinal research approach

A longitudinal research approach was employed by the involved researchers (Yin 1994) for examining the impacts of the Resilience Lab. Table 1 summarizes the activities in the different phases of the research in the Resilience Lab, the methods and stakeholder engagement activities. The Resilience Lab included four different types of actors involved in the research activities (based on a stakeholder analysis): (1) members and practitioners of the Resilience Lab-consortium, (2) lab participants like volunteers, children, families, teachers, inhabitants of Carnisse and Charlois district, (3) neighborhood professionals like welfare workers, civil servants, policy makers, youth coaches, social workers, urban experts and professionals and (4) local actors from other districts. This distinction in actors is relevant for understanding the range of perspectives respondents have regarding place meanings. A detailed description of the research activities per phase of research corresponding to Table 1 is given in the Supplementary document.

**Table 1:** Methodological architecture and overview of activities

	Phase of the Veerkracht Lab	Action research activities	Monitoring and evaluation activities
Pre 2011	Scoping phase	–	2 meetings on conceptualization of Resilience Lab 1 lobby meeting with the municipality setting up the Lab
09.2011–02.2012	Setting the scene	–	3 progress meetings with lab practitioners on the conceptualization of Resilience Lab, 2–3 h long per session 56 interviews with residents, civil servants, local entrepreneurs, etc 21 of participants’ observations during neighborhood meetings Desk research on Carnisse, and on neighborhood discourse in Rotterdam
02.2012–06.2013	Mobilization and place making	8 participatory workshops, 2-h each with 10–15 participants from the area 5 participatory workshops related to the reopening of a community center (in total 48 participants) 5 participatory observations within local activism (focus on community center)	23 of interviews with residents, civil servants, local entrepreneurs, etc 18 of participants observations during neighborhood meetings 12 monthly progress meetings of the Veerkracht lab, 1.5 h long Developed a monitoring framework
06.2013–12.2015	Deepening, broadening and upscaling	10 of participatory observations within local activism with a focus on the community center 63 of participatory observations within local activism with a focus on the community garden 25 of participatory observations within several experiments (e.g., the neighborhood guide, the Community Bond Carnisse, etc.)	45 of interviews with residents, civil servants, local entrepreneurs, etc. 38 of participants’ observations during neighborhood meetings 17 monthly progress meetings of the Resilience lab, 1,5 h long 7 monitoring sessions with lab practitioners, 2–3 h long per session 3 monitoring sessions with initiators of reopening the community center 2 participatory workshops on impact of public places and networks in Carnisse (approx. 50 participants in total) Writing a yearly progress report
02.2015–12.2015	Evaluation and transferability	–	34 interviews with different actors in the neighborhood 4 monitoring sessions with lab practitioners, 2–3 h long per session 1 participatory workshop on impact of Resilience Lab with participants, residents, etc., 2 h long (approx. 20 participants) Writing an evaluation report on the Resilience Lab

## Results of place making in Carnisse

Using the conceptual lens developed, we analyze the case study results by identifying (1) the new social relations established in the area, (2) the new narrative of place, ‘Blossoming Carnisse’, and (3) the established symbolic places that were iconic for moving the transition forward. In this way, we explore how experimentation in the Resilience Lab creates new sense of place and enables urban sustainability transitions.

### New relations between people and between people and place in Carnisse

The main element of experimentation in the Resilience Lab was the application of the different engagement and participatory methodologies for establishing new forms of collaborations between citizens and the city, and new social relations between the residents as well. The partners and participants involved in the Resilience Lab addressed it as ‘an experimental program’ within its spatial and administrative boundaries, with the focus to test new methods and practices on discovering (and co-creating) urban regeneration solutions or approaches. They discerned four different ‘fields of interaction’: home, school, outdoors and neighborhood. Each of these fields consisted of individuals, networks and institutions on which the activities were focused. By working together in the Resilience Lab, the aim was to increase the interaction between the different target groups via the engagement in these four fields of interaction and to discover/create more integral ways of working for transformation at the scale of an urban neighborhood. The working assumption of the Resilience Lab was that interactions took place via collaboration in practice and produced multi-faceted added value (financial, social and ecological).

Initiating the core activities helped increase visibility and trust of the Resilience Lab in the neighborhood, although competition and mistrust were still tangible. Some activities were seen as an add-on to current activities in the neighborhood, e.g., a participatory process focusing on the future quality of life and primary school activities. Other activities were more welcomed such as an intervention supporting local change agents to reopen the community center in a cooperative manner.

However, it was in the actual physical activities where different networks (of residents, practitioners of the Resilience Lab, civil servants, etc.) would meet and interact with each other. The two gardens, the three primary schools in Carnisse, the community center, residential homes and residential streets proved to be central for facilitating new and existing relationships, not only in a professional sense, but also—and maybe predominantly—on a personal level. For instance, different cultural and ethnic groups blended in language courses, sewing classes and agricultural workshops. But also, different age groups were mixed in cooking programs and festivities in the neighborhood center and at schools. And neighbors who never spoke to each other came together in greening their street, where they helped each other by planting flowers, shared a lunch and/or had a coffee. Interaction was, however, not always friendly and warm, since gossip, slander, and tensions were also part of working and living together in Carnisse (see “Symbolic places in Carnisse”). But also the physical presence of professionals and volunteers at a certain site and certain time created a needed sense of structure in the tumultuous life in Carnisse.

These sites proved to be central in promoting collaboration between different parties and networks, since they were the places where the different target groups interacted and collaborated in shared activities. Most of these collaborations were fuelled by a mutual interest in working together as to reach corresponding goals, which proved to be crucial to invest in longer term collaborations. Respondents stated that these collaborations are built on reciprocity and trust. In the 4 years of the Resilience Lab, different types of collaborations flourished: short-term and long-term; incidental and structural; one-sided (or ‘parasitic’) and reciprocal. It proved to be a challenge for the people and networks in Carnisse to create structural collaborations with each other, since people perceived an erosion of informal and formal networks. Residents moved relatively quickly to other parts of Rotterdam or outside the city (e.g., in 2011 59.6% of the residents moved within 5 years of living in Carnisse). Due to welfare reforms, professionals were frequently laid-off, did not get extensions of contacts or were transferred to other districts. Therefore, respondents questioned the long-term endurance of the collaborations, including the ability of primary school teachers, volunteers, and community workers to continue the activities of the Resilience Lab on their own and with their own funding.

Relations and networks were not limited to administrative borders of Carnisse. These borders proved rather fluid in both the conceptions of Carnisse itself as the relations and networks present. For example, children at primary schools in Carnisse often lived in other neighborhoods, and volunteers at the garden lived in other cities, or, villages nearby Rotterdam. People engaged in networks in Carnisse lived outside the administrative boundaries, but felt more ‘at home’ there than in their own neighborhood. Professionals engaged in Carnisse are active in other parts of Rotterdam and often do not live in Carnisse or the Southern part of Rotterdam themselves (some do not even live in Rotterdam). Place attachment, meanings, networks and relationships transcend the administrative boundaries of Carnisse.

### A new narrative of place: “Blossoming Carnisse” in the year 2030

With the experimentation process in the Resilience Lab, a new narrative of change was created and summarized in the form of a future vision and the operating guiding principles of the Resilience Lab. The focus on the connection of people and places as “the starting point” for “learning infrastructures” in the area revealed the shift from a reductionist understanding of place to a socially mediating facility for change and development.

In 2012–2013 action researchers facilitated a community arena process that focused on envisioning a sustainable future of Carnisse. The central question was: “what does living entail in the year 2030 for a resident in Carnisse?”. A group of residents, entrepreneurs and professionals held 8 sessions in which they negotiated problem perceptions and shared meanings; some of which varied widely and even conflicted between groups. An outcome of the complementary field interviews was the coexistence of conflicting views on the neighborhood by policy actors who viewed the place with a stigma of a deprived neighborhood that requires extra policy attention versus the lived experience view of residents who expressed that there was nothing wrong with their neighborhood. Respondents holding the latter view were eager to highlight positive aspects, e.g., it is youthful and diverse population, and it is a nice, quiet, central location in the city. The policy interventions were discussed together with the weariness that people felt about participating in these processes and the erosion of institutional networks in the neighborhood due to severe budget cuts that led to closing of several public facilities (e.g., two community centers and the educational garden). The result of this process was a shared vision called ‘Blossoming Carnisse’ that included several transition pathways for the future, an agenda for transformative and experimental actions. It connected the expressed aspirations to initiatives already happening in Carnisse while also critiquing current neighborhood dynamics.

Another discursive turn was made in the Resilience Lab itself, where the practitioners and members tried to connect to an alternative discourse within urban planning in the Netherlands more broadly. This discourse focused on a proclaimed growing social movement of inhabitants and social entrepreneurs trying to reclaim public spaces and engaging in innovative practices (e.g., urban gardening, fostering community bonds, local currencies, co-creation of public squares and self-maintenance of community buildings). These trends were based on alternative paradigms and according to the Resilience Lab partners, appeared to be well-suited to current socio-economic needs of the community. In trying to distinguish the Resilience Lab from the status quo in neighborhood development, it drew up five guiding operating principles (during monitoring sessions) in 2012–2013:

- Strengthening and utilizing the self-organizing capacity of its people fosters the resilience of Carnisse. It is about talking with people instead of talking about people.
- People and places are the starting points: from here ‘learning infrastructures’ are built, guided by the daily routines and lifelines of individuals and their networks.
- Methods and activities are developed in an organic manner in the Resilience Lab to fit the needs of the area, i.e., from a practical and operational rationale.
- Connections and collaborations (on several levels) are sought based on innovation and reciprocity. This implies less pressure of bureaucratic control, rules and procedures.
- Participants strive for a balance between top–down intervention and bottom–up self-organization.

In all the operating guiding principles, the notion of collaborative governance demonstrates that reciprocity and institutional connections are key for escaping stigmatization of the place and its people. The Resilience Lab and the narrative of change it co-created, helped establish a connection between the context of urban regeneration processes at the city level and its local social innovation processes. The Resilience Lab facilitated a dialogue about perceptions on the present and the future of the neighborhood, its problems and their origins.

The Resilience Lab opened an opportunity to discuss and negotiate the different meanings of Carnisse. One prime example was the envisioning process that drew from the historical roots of the neighborhood. By highlighting Carnisse as a place that is constantly changing and at the same time seemingly remaining constant over decades, it opened the notion of transformation. During the envisioning process, alternative (un)sustainable futures were also discussed, e.g., a ‘Bleeding Carnisse’ where Carnisse transformed into a ghetto. This vision contributed to the awareness of the possibility and drivers of change. Dominant actors like the district council stressed that due to the absence of housing corporations and physical investments not much could be changed in the coming years. When these actors followed the dominant narrative of a deprived neighborhood, residents and neighborhood professionals were offended by and countered this notion. A side effect of the Resilience Lab was that discussions about Carnisse with local communities increased the salience of the place around the administrative bounds of the project. Where residents did not exactly know where the administrative boundaries of the neighborhood started and ended, Carnisse became increasingly demarcated as an administrative ‘space’. This fixation was overlooked by civil servants who consider these administrative borders for granted and as generally known. To summarize, through envisioning, a common understanding and meaning transcending the different levels of ownership of the place and connectedness to it was created. The importance of living and working together as a community with ties to each other and to place was a center notion in the vision.

### Symbolic places in Carnisse

The neighborhood of Carnisse was flawed, in the sense that it was not a common place most people could identify with and have shared meanings and experiences. Carnisse proved to be a somewhat amorphous signifier. As stated before, Carnisse was fixed for the actors who participated in more formal institutions and networks. For most of the residents and volunteers, however, Carnisse—and their sense of belonging and ownership—was more centered around certain public and private places in their living environment, like their home, certain shops, and public facilities like sport venues and community centers. These places commonly crossed the official borders of Carnisse and were part of another neighborhood. Therefore, the focus of the Resilience Lab shifted accordingly. For instance, the envisioning process was directly connected to the reopening of a closed community center, making it a symbol for change and an alternative future, and in this way, tapping into the transformative potential of these public places early on.

During the Resilience Lab, different places were revitalized like the community center (the Heart of Carnisse), two community gardens (the Carnisse garden and the Tennis garden), some family homes and three primary schools. These were places of encounter in the neighborhood and bottom–up collective practices were set up to collectively transform the relations between people, their place and their everyday routines. These different places were reestablished to manifest symbolic meanings in the area like the resilience of its citizens and an alternative future for Carnisse. In the sections below we highlight two of these ‘symbolic places’: the community center and the community garden. Both places have been reclaimed as a sort of counter movement by the local communities and transformed into more inclusive, open and broadly supported places.

#### Community Center

Several community centers were closed-down due to budget cuts in 2010 and 2011, one them being Arend & Zeemeeuw that had a long tradition in Carnisse and Rotterdam. It was a symbol for the (historical) connectedness of residents with Carnisse, and it became a symbol for transition and resistance during 2012–2016, as residents and local entrepreneurs started a petition, occupied the building and reclaimed the center by running it in a self-sufficient manner. In 2012, the Resilience Lab played an active role in facilitating the action group and coordinated actions between different parties for reopening the community center. As of 2013, they reopened the center by organizing monitoring sessions for ongoing activities and conducting interviews and participants’ observations to map needs for the center to consider.

Relatedly, many activities were initiated by local communities like dancing workshops, flower workshops, educational activities, sewing classes, music workshops, religious events, parties, and games. This soon became a ‘flagship project’ for the ‘participation society’, and was celebrated by public officials and politicians. It led to several conflicts with the local municipality and within the action group. The municipality perceived the ‘action group’ as protesting against them and refused to back the group in reopening the community center (e.g., by asking for commercial rent prices). Within the action group, a conflict arose about how to reopen the center and who was in charge. In short, there was a group that wanted to transform the center into a welcoming place for all residents and to professionalize the its management and another group that wanted to keep the center as it was and run it via volunteering and subsidies. This conflict escalated and led to a departure of the latter group who was forced out (also because of pressure from the municipality). During the reopening in June 2013, the center was renamed as ‘The Heart of Carnisse’. In the following years, the center proved to be a meeting point of local communities (religious, sports, primary schools, child daycare, migrants, etc.). As to date, the center continues to have a somewhat conflictual relationship with the municipality with a constant threat of closing-down.

#### Community garden

**Fig. 2:**
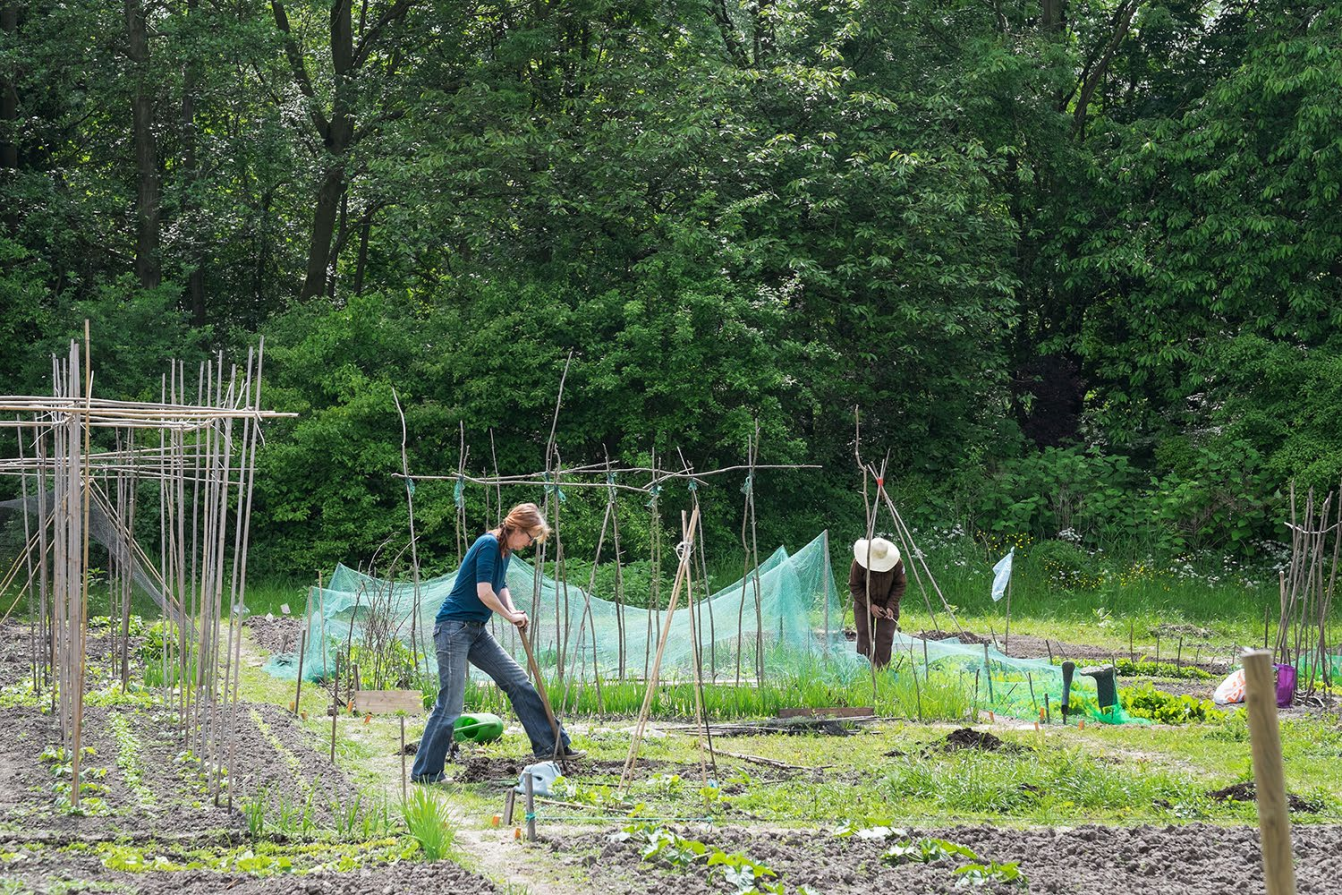
Symbolic places. Gardeners who are literally place making by putting their shovel and hands in the earth. This picture features the Carnisse garden, a community garden which was reopened by the Resilience Lab together with local residents and volunteers

The professionally led educational garden was shut down in 2012 due to budget cuts from the local municipality. One of the partners in the Resilience Lab assisted the residents and volunteers in transforming this closed-off garden into a community garden, the Carnisse garden. In 3 years, the garden was transformed from an anonymous, shut-off place to an inclusive, open and broadly supported place. Crops, herbs and flowers were cultivated by and for the residents. These were traded to those who helped out with the garden (guiding principle of reciprocity) and were given away to people in need (in shelters, food banks, etc.). Primary schools organized educational activities, elderly homes organized activities on the garden, and ex-addicts were helping in the garden and in return, got vegetables to cook with for their shelters. The number of visitors, volunteers and collaborating organizations in the neighborhood (and the city) grew extensively (Fig. 2). It became a ‘flagship project’ and was portrayed in several studies and celebrated in media coverages. However, in 2015, it was shut down by the local municipality, because the ground was sold to a project developer who wanted to build a parking lot and buildings on it. During the closure in 2011, a petition was started which led to about 100 signatures, but in 2015 the action group (consisted of residents and volunteers) collected more than 2100 signatures. This led to escalating events in the end of 2015 that were discussed in the city council by political parties, several aldermen and the highest ranks of the administrative body of service department directors. Due to bureaucratic and procedural difficulties, the garden could not be saved, but because of a resolution in the city council, a substitute garden was to be created and cultivated.

We contend that both the community center and the garden functioned as symbols for the desired place, since they showcased and strengthened the connectedness of local communities with the places, and networks of residents and professionals were created to nurture them and later to prevent them from closure. This comes in agreement with research on community gardens that confirm that they require community effort to be established, cultivated and remain productive spaces. They require leadership for designing and deciding the type of cultivation (plot or mixed), whether the plants will be edible or not and what the accessibility of the garden will be. Community gardens are places for establishing community connections via greening (Tidball and Krasny 2014) and sites of strengthening social–ecological resilience. Okvat and Zautra (2014, p. 83) note that “positive engagement through gardening, beautification, and community organization reflects hope for better conditions and the agency to bring these conditions about, which might be quite important to demoralised or disenfranchised people in a disaster zone”; relevant for impoverished neighborhoods.

## Discussion

### Does experimentation contribute to creating new sense of place in urban settings, e.g., neighborhoods?

The Resilience Lab aimed at strengthening the resilience of the neighborhood, empowering children, families and communities. While achieving these, the Resilience Lab achieved identifying new ways of neighborhood development, questioned local democracy, and power relations, established new networks, and innovating practices. The experimentation in the Resilience Lab contributed in creating new sense of place by establishing new meanings of place, a transformative place vision of ‘Blossoming Carnisse’ and the strengthening of relations between people and their neighborhood as well as the creation of new relations within and across the community.

Creating new meanings of place through experimentation further motivated the sense of change from within the community. The new meanings, even when contested between community participants, contrasted with the place meanings of the past and present and mobilized thinking and doing to pursue the new meanings of place. The community’s sense of place was captured in the shift of framings of meanings ‘from a stigmatized to a blossoming place’.

The transformative vision combined with the symbolic places and alternative practices, created a narrative of place which in turn, contributed to creating a place identity and increased the sense of community. As such, creating sense of place increased community agency that was empowered and self-directed to act to achieve the vision. The visible and tangible transformation of place came from establishing and reclaiming the symbolic places (the Community Center and the Community Garden) that shifted the discussion of place from further degenerating to becoming a welcoming, growing, ‘blossoming’ place; enriching in this way the experience of place. The community contrasted the under-recognized importance of these places in the past with the stewardship they received as places of collective meaning. The symbolic places contributed to a place transition towards a ‘thick place’, “places (…) made as much as they are discovered and (…) made in and of affect and practice.” (Duff 2010, p. 882).

### How does creating new place meaning enable urban sustainability transitions?

Place becomes an attractor and a medium for transitions. Place becomes an attractor for transitions by mobilizing changes in ways of thinking, acting, organizing and relating. It specifically does so by, viewing how place itself changes in meanings, attachment, in physical characteristics and in the relationships established between place and people. Place also functions as a medium and mechanism of change; implying that for governing urban sustainability transitions the place-based characteristics of transitions need to be examined and understood next to the normative orientation of sustainability.

The way place changes throughout the experiment entail a visible transformation of the neighborhood. We see place as a critical component in addressing sustainability challenges and place to be the site of transitions. A place-based focus allows people to address the sustainability challenges and to be part of the transition in the making.

A dynamic understanding of place is pivotal for positioning experimentation as a means to facilitate transformative sense of place and to instigate urban sustainability transitions. Connecting the vision and symbolic understanding of place with the places where new relations and new identities were formed is critical for moving the transition and specifically for a tactical implementation of the transition agenda at local places. A dynamic understanding of place as a theme or starting point of experimentation comes also in line with an understanding that cities are “transitionscapes” and as such, open and susceptible to continuous change and innovation (Frantzeskaki et al. 2017).

**Fig. 3:**
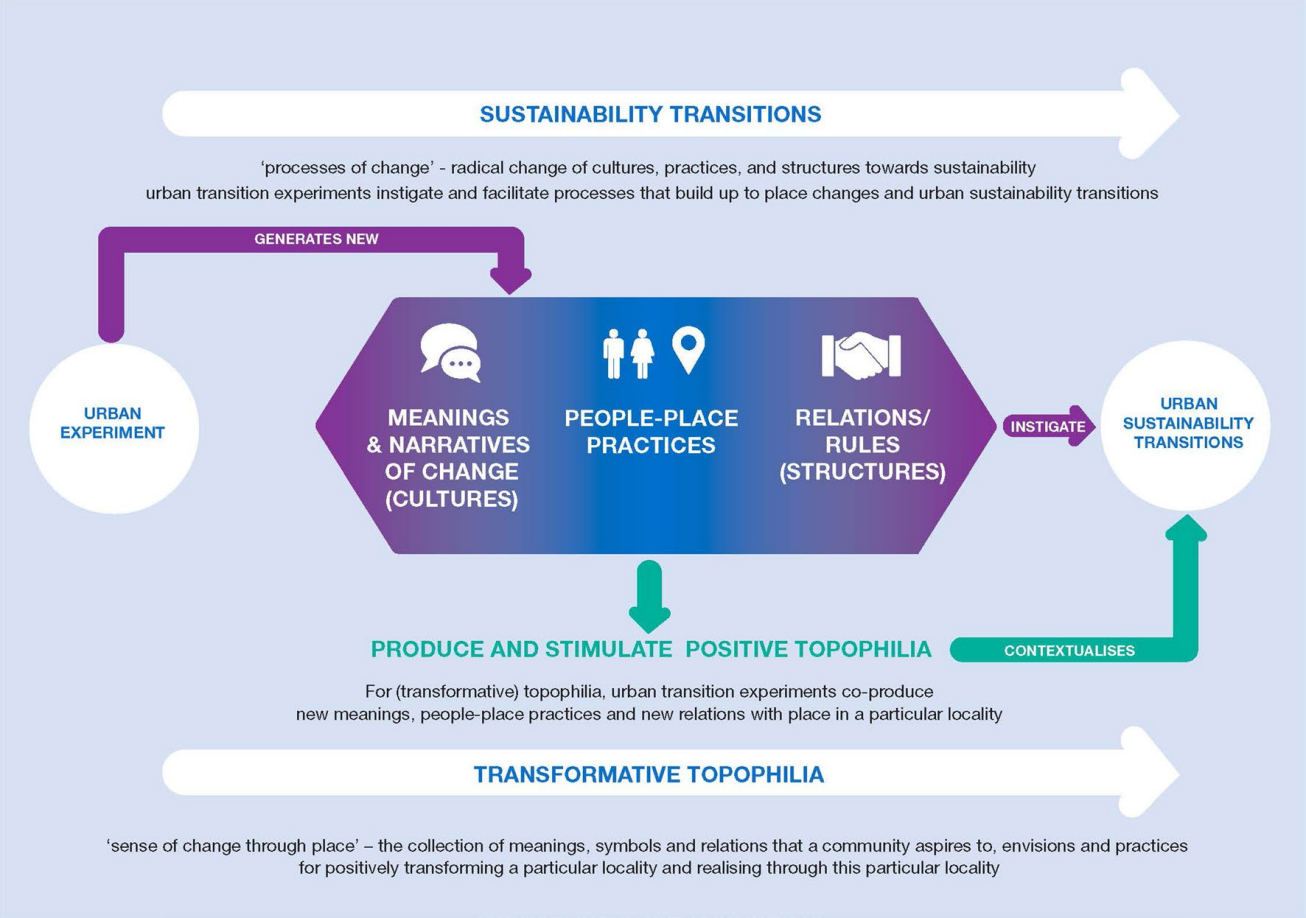
Sense of place and experimentation. A conceptual map of the relations between sense of place and experimentation for urban sustainability transitions

Conclusively, drawing from the case study of the Resilience Lab, we contend that urban living labs (as a format of urban transition experiments) that are place-explicit can connect a sense of change (transformation) with a sense of continuity by co-creating new narratives of place, co-producing knowledge on new practices and new relations between people and place and by allowing the co-design or (re)establishment of places with symbolic meaning. As thus, they enable urban sustainability transitions (Fig. 3).

### What are the implications and design characteristics for experimentation to contribute to urban sustainability transitions that are place-embedded (through creating sense of place)?

By addressing the notion of place-embeddedness, we try to address different kinds of impacts of urban experimentation and respond to the third research question. We arrive to four lessons derived from the analysis of the case that contribute to the scholarly work on urban experimentation, urban transitions and sense of place. In doing so we also open an array of further discussions and research questions as well as the necessity for more comparative research on place-based urban experimentation.

First, experimentation delivers social outcomes rather than simply the technological visions so prominently emphasized in sustainability transitions studies. What we observed in the Resilience Lab is that it created a sense of place that transformed the urban neighborhood via strengthening the ties of its community to the place. Varying place meanings between different actors, e.g., government that embraces the ‘participatory society’ (Dutch equivalent of the ‘Big Society’) and residents that see public places and facilities being closed (and sold) manifest competing meanings that embody a power dimension (Stedman and Ingalls 2014). Experimentation not only reveals these contestations but also dissects the power dynamics of place narratives and meanings, opening the discussion ‘sense place for whom’ and ‘for what kind of transition’ we use experimentation. In this way, experimentation contributes to social outcomes through creating new social relations and new relations between place and people.

Second, experimentation can also be a process to establish agentic processes. Through establishing new sense of place (e.g., via place meanings), creating transformative agency is possible given that, symbolic meanings strengthen ties in the community and can mobilize action to transform the place into the place imagined/aspired to. Experimentation objectives were set more on learning and processes of empowering rather than trialing nor diffusing social or technological innovations. So far, research has understood experiments as not agentic configurations but rather ‘systemic’ configurations of protected spaces for innovation to be trialed, nurtured and grow, thus conditioning diffusion and mainstreaming of innovations. In this case, however, we see that experiments are agentic, create narratives of new meanings, narratives of place change and create feedback loops between meanings, places and action for change.

Third, experimentation does not take place in a vacuum, since it is embedded in a certain context where (trans)local discourses have a significant influence on the dominant institutional setting, meanings and narratives in place. Experimentation, therefore, needs to be connected to dominant discourses of ‘the local’ (like the Resilience Lab is for instance embedded in strategies aimed at ‘urban deprived neighborhoods’), because only then the experiment can explicate how it relates to and impacts the status quo through triggering alternative narratives, meanings, relations and symbolic places. Hence experimentation, from a transitions perspective, is not only just about place making, but also about challenging the dominant discourses and practices in their context.

Fourth, an implication of the above is that experimentation can be instrumentalised to manipulate positive sense of place. In the case of Carnisse, experimentation was employed as a last resort when many regeneration interventions deemed ineffective. However, we may see the risk of using experimental approaches to make ‘place branding’ schemes socially acceptable, “manipulating (…) to create a positive sense of place” (Cleave et al. 2016) with the side effect of a gentrification of newly branded neighborhoods. With the evidence of our case over and our knowledge from researching this area over the past 5 years, we contend that for meaningful transformations of place, place meanings in particular, need to be mediated and facilitated (e.g., with experimentation) neither forced upon nor manipulated. Inclusive, open and socially reflexive processes of experimentation can be the means for meaningful sense of place (meanings that are associated with attachment) that enables deeper and lasting social transformations.

## Conclusions

Our paper is the first attempt in bridging the scholarships of sense of place and sustainability transitions, with the aim to go beyond the trivial argument that ‘place matters for sustainability transitions’ to addressing what a place-based perspective and understanding contributes to making sense and design interventions for governance of urban sustainability transitions. With the evidence from our case study and the conceptual bridging of the two scholarships, we contend that place becomes an attractor and a medium for sustainability transitions.

We have shown that experimenting is one important way to address sustainability transitions, and not only a means to instigate or catalyze them per se. Through experimentation, local actors explore alternative narratives, meanings, and relations and address transitions-ideally those that will result in deepened attachment through the creation of preferred visions or meanings—in a certain place. Urban living labs like the Resilience Lab are not trying to realize a certain regime shift in a societal (sub)system, since the level of a neighborhood is much too limited for this (since transitions are also apparent on different scales, e.g., city, regional, national and global scale). Thus, the impact of experimentation is not to be seen in the level of impact on (a certain) transition (e.g., energy transition), but it is rather about learning on what needs to change, how it can be changed and what one’s own role is in this change process. Experimentation, thus becomes a process of awareness for thinking and acting for transformative change and more of a process of contextualizing transitions, rather than shifting a certain regime.

For future research, we propose conceptual and empirical examinations on how place meanings and attachment contribute and relate to urban sustainability transitions, to the means for their governance including experimentation and to new planning approaches. In this case study, we observed the sense of place to be created through experimentation. This was made possible due to the longitudinal research approach that allowed us to observe and examine the change in narratives, place making and place changing. It was made evident that a long-term research horizon was crucial for examining in-depth slow-social processes of transformation such as the creation of symbolic places, the evolution of social relations and the trust building required for reciprocity and partnerships. We thus propose that comparative and in-depth case studies could examine the relation between place and transitions, to further employ longitudinal approaches on this topic. Another topic relevant to this direction may be the exploration and assessment of durable effects in creating sense of place post-experimentation. Lastly, future work could explore the different governance means required to maintain and foment the transformative capacity of urban neighborhoods as sites where transitions can be initiated and connected.

## Electronic supplementary material

Below is the link to the electronic supplementary material.

Supplementary material 1 (DOCX 168 KB)
