# Supplementary material for: Sense of place and experimentation in urban sustainability transitions: the Resilience Lab in Carnisse, Rotterdam, The Netherlands
Source: Sustain Sci. 2018 Apr 27;13(4):1045–59. doi: 10.1007/s11625-018-0562-5 (PMC6086306; doi:10.1007/s11625-018-0562-5)
Supplement: Supplementary file 1 — Supplementary material 1 (DOCX 168 KB) [file 11625_2018_562_MOESM1_ESM.docx]

**Supplementary Material**

**Detailed analysis of the longitudinal research approach in the Veerkracht Lab**

A longitudinal research approach was employed by the involved researchers for examining the impacts of the Veerkracht Lab. Table 1 below summarizes the activities in the different phases of the research in the Veerkracht Lab, the methods and stakeholder engagement activities. Stakeholder analysis included roughly five different kind of actors involved in the research activities: 1) members and practitioners of the Veerkracht-consortium, 2) participants of the lab like volunteers, children, families, teachers, inhabitants of Carnisse and Charlois district, 3) neighborhood professionals like welfare workers, civil servants, policy makers, youth coaches, social workers, urban experts and professionals and 4) local actors from other districts. This distinction in actors is relevant for understanding the range of perspectives respondents have regarding place meanings. A detailed description of the research activities per phase of research corresponding to Table 1 is given here as Supplementary material.

The researchers involved in the Veerkracht Lab had two type of research activities: more classic monitoring and evaluation activities and action research activities. The action research activities were predominantly aimed at implementing a so-called Community arena process (Wittmayer 2016), based on the principles of transition management (Frantzeskaki et al 2012) and adapted to the local context of communities. This method resulted in a process of a more deliberate nature such as engaging in discussions about current problems in Carnisse and developing a vision for the future of Carnisse with corresponding images and pathways to realize this vision. This was followed by activities of a more practical nature: experiments to learn about how to reach a long-term goal. The deliberative process was focused on co-creating the vision a ‘Blossoming Carnisse’. This vision was formulated in 8 participatory workshops with 21 participants from the area and subsequently broadened in the neighborhood, e.g. during an official Inhabitants Forum with approximately 150 attendants. The process of experimenting revolved around the reopening of the community center where researchers played a mobilizing role by organizing 5 participatory workshops before eventually handing it over to residents and professionals from Carnisse in October 2012. In the next years, researchers remained connected to the center by participative observations and organizing three monitoring sessions with the initiators. Next to the experiment of reopening the community center, another key experiment from the Veerkracht Lab was researched with participatory observations: the community garden (‘the Carnisse Garden’) which was revitalized in 2012-2015 and eventually shut down in 2015. Other smaller experiments in which researchers participated were the startup of a long-term neighborhood-based internship (the ‘Neighborhood Guide Carnisse’) and of an innovative investment scheme for neighborhood development (the ‘Community Bond Carnisse’).

Activities in this monitoring-and evaluation process were writing a yearly progress report and evaluating the Veerkracht Lab. For collecting data, all partners in the consortium had to deliver their results each year in a quantitative way (e.g. how many people participated in which activity). Their main method of data gathering was in a qualitative way. During the scoping phase the role of the researchers was limited to the attendance of several progress meetings (approximately 3). In the period of September 2011 to August 2015 a total of 158 qualitative interviews were held. These focused on several categories of questions, e.g.: personal questions and field of interest, questions about Carnisse, questions on practices, and questions about specific collaborations with(in) the Veerkracht Lab. The interviews were conducted by four different researchers. In this same period, 77 participatory observations and field visits were carried out and 33 monthly progress meetings of the Veerkracht Lab were attended by the researchers. In the monthly progress meetings, the consortium exchanged updates and activities in the field within an informal setting (about 5 – 7 people were present per meeting). These participatory observations and field visits aimed to observe and examine the different practices within the lab, and varied from attending judo-classes at primary schools, cooking-clinics on the garden, educational workshops for volunteers, political debates about the community center and garden, and neighborhood lunches. These visits and observations were complementary to the other research activities to get a full scope of the design, practices and processes of the Lab. Researchers co-organized 11 monitoring sessions with Lab members and practitioners. These sessions were aimed at identifying crucial adjustments to the Lab and identifying lessons learned from the past weeks, months, and years. Next to these activities the researchers organized 3 participatory workshops with the different involved actors mentioned above. These workshops were aimed at discussing the process and impact of the Lab with about 25 people from all actor-categories described above and focused on the sustainability of interventions and practices. Researchers carried out an extensive desk-study (collecting academic and grey literature to map the discourse of current debates in welfare reform and neighborhood development). The result was a rich set of data that related to different perspectives on life in Carnisse from all the different actors involved.
